# The reimplantation valve-sparing aortic root replacement technique for patients with Marfan syndrome: A single-center experience

**DOI:** 10.1038/s41598-019-48572-9

**Published:** 2019-08-19

**Authors:** Jamila Kremer, Mina Farag, Marcin Zaradzki, Gabor Szabó, Arjang Ruhparwar, Klaus Kallenbach, Matthias Karck, Rawa Arif

**Affiliations:** 1 Marfan Center University Hospital Heidelberg, Department of Cardiac Surgery, University Hospital Heidelberg, Heidelberg, Germany; 2 INCCI HaerzZenter, Department of Cardiac Surgery, Luxembourg City, Luxembourg

**Keywords:** Outcomes research, Risk factors

## Abstract

Valve-sparing aortic root replacement (VSARR) through reimplantation technique is widely regarded as optimal surgical approach for Marfan syndrome (MFS) patients. Perioperative and long-term data from all MFS patients undergoing VSARR using David’s technique at our center from 2007–2018 were analyzed. We included 56 patients with a mean age of 32.3 ± 12.3 years. Logistic EuroSCORE was 7.96 ± 5.2. Among others concomitant surgical procedures included aortic arch surgery (8.9%), mitral valve repair (23.2%) and replacement (1.7%). There were no operative deaths, nor in-hospital-mortality. One patient underwent re-exploration for bleeding, dialysis and pacemaker implantation was required in one case each. There was no occurrence of low-output syndrome nor neurological complications. Significant gender differences were not found, except for intraoperative blood transfusion occurring significantly more often in the female gender (p = 0.009). Despite significantly longer procedural times, concomitant surgery did not negatively impact overall outcome. Freedom of reoperation of the aortic root was 100% at 1 year, 97.7% at 8 years. Until last follow-up (61 ± 38 month) all patients survived, with no evidence of endocarditis. We emphasize once more that VSARR using David’s procedure is a safe method for MFS patients with excellent long-term results even if concomitant procedures are performed.

## Introduction

Mutation of the fibrillin-1 gene (FBN1) leading to defective extracellular microfibrils results in divergence and instability of the entire connective tissue^1^. In patients suffering from Marfan syndrome (MFS) the FBN1 mutation is inherited autosomal dominant with considerable phenotypical variability. The skeletal system, eyes, dura, skin and the pulmonary as well as the cardiovascular system are affected. Although MFS is a systemic disorder, reduced life expectancy is primarily determined by the extent of cardiovascular manifestations^2^. Dilation of the aortic root is a result of increased aortic stiffness and pulse wave velocity, enhanced by fibrillin fragmentation due to the underlying FBN1 mutation^1^.

The aortic valve-sparing reimplantation technique first described by David and Feindel^3^ was originally developed as an aortic valve-sparing operation for patients with aortic valve incompetence and aneurysm of the ascending aorta^4^. Today, prophylactic replacement of the dilated aortic root with preservation of the native aortic valve leads to excellent results, allowing an almost normal life-expectancy, avoiding the need of lifelong anticoagulation, especially for younger patients with MFS^5–8^.

Our study assesses outcome in patients with MFS operated on exclusively using the aortic valve-sparing reimplantation technique for aortic root aneurysms during more than a decade at a single center.

## Patients and Methods

From March 2007 to December 2018, 229 patients underwent valve-sparing aortic root replacement (VSARR) at our department using the aortic valve-sparing reimplantation technique by David^3^, of whom 56 patients were diagnosed with MFS according to the modified Ghent criteria^2^. Patient data were prospectively collected from our hospital database and MFS outpatient clinic. Informed consent was obtained from all individual participants or their legal guardians included in the study. Follow-up was obtained after consent and approval of our institutional review board (Ethics committee University of Heidelberg). All research was performed in accordance with the ethical standards of the institutional committee and with the 1964 Helsinki declaration and its later amendments. All 56 surviving patients (100%) were either seen as outpatients after discharge or were contacted to obtain postoperative follow-up.

One patient was excluded from our study, who underwent aortic root replacement with a mechanical valve prosthesis due to failed VSARR, in whom a Valsalva graft had been used.

### Surgical technique

All patients, but one, were operated via median sternotomy with use of cardiopulmonary bypass (CPB). One patient was operated on by left parasternal thoracotomy due to severe pectus excavatum. Standard central cannulation of the ascending aorta and the right atrium were performed in 43 cases (76.7%). If indicated, venous cannulation was established by separate venae cava superior and inferior cannulation in order to perform concomitant mitral valve repair (13 patients, 23.2%) and replacement (one patient) or patent foramen ovale closure (5 patients, 8.9%). Mean core cooling was accomplished at mild hypothermia with 34 °C rectal temperature. Five patients (8.9%) underwent concomitant aortic arch procedure, therefore mean hypothermia in these selected cases was 24.3 °C. Cardiac arrest was induced by cold Bretschneider’s solution (Custodiol, Dr. Franz Köhler Chemie GmbH, Bensheim, Germany).

After incision of the ascending aorta, the aneurysmal portion was excised as well as the sinuses of Valsalva. The aortic valve leaflets were then reimplanted inside the vascular Dacron graft. The coronary arteries were also reimplanted through button incisions in the vascular prosthesis. For concomitant aortic arch surgery, circulatory arrest was established by discontinuation of CPB. Selective antegrade cerebral perfusion was initiated through cannulation of the left common carotid artery and the brachiocephalic trunk. Perfusion volume was 8–12% of calculated cardiac output and perfusion pressures was held between 60–80 mmHg. Proximal graft-to-graft or graft to aortic anastomosis at various levels completed the repair.

Plication of the aortic valve had been performed in two patients, of whom one presented with bicuspid valve. Mitral valve repair was performed in 13 patients using Carpentier-Edwards Physio II (Edwards Lifesciences Corp., Irvine, USA) annuloplasty ring in all cases. Triangular resection and sliding plastic of the posterior leaflet was performed in two patients and cleft repair in another two. All other repairs (n = 9) were restricted to annuloplasty due to annular dilation. Sole patent foramen closure and chest wall repair had not been considered as relevant concomitant procedures for statistical analysis. Mitral valve intervention and surgery of the aortic arch had been considered as concomitant procedures for statistical analysis (n = 19, 34%).

### Follow-up

Postoperative computed tomography angiograms (CTA) and magnetic resonance angiographies (MRA) were analyzed during standard follow-up visits at our MFS outpatient clinic. During these visits, echocardiography was routinely performed to evaluate postoperative aortic valve function. Patients who were seen in their referring hospital or by the referring cardiologist, were contacted to forward their recent follow-up data.

### Statistical analysis

Statistical analysis was performed using IBM SPSS Statistics version 25 software (SPSS, Chicago, IL). Normally distributed continuous variables were reported as mean ± standard deviation. Categorical variables were reported as frequencies and percentages. To elaborate gender differences or differences of patients undergoing concomitant on-pump procedures (n = 19) preoperative, operative and postoperative data were analyzed by Mann-Whitney-U-test and 𝜒2 test. Freedom of aortic root intervention was estimated using the Kaplan-Meier method. Due to complete survival, no survival estimation was performed. Independent risk factors were analyzed by Cox regression analysis considering secondary interventions of the downstream aorta.

## Results

Demographic data are given in Table 1. Mean age at the time of VSARR was 32.3 ± 12.3 years with no gender differences (p = 0.191). Thirty-five patients (62.5%) were male. Mean diameter of aortic root aneurysm was 5.0 ± 0.6 cm. Thirty-three patients (58.9%) presented with aortic regurgitation (AR) before VSARR.

**Table 1:** Preoperative data.

patients	56
male	35 (62.5)
age, years (range)	32.34 ± 12.35 (12–60)
height, cm	188 ± 10
weight, cm	80 ± 18
BMI, kg/m2	22.3 ± 4.6
emergency	2 (3.6)
re-operation	4 (7.1)
acute aortic dissection Typ A	2 (3.6)
chronic aortic dissection Typ B	4 (7.1)
aortic regurgitation:	
I	19 (34)
II	8 (14)
III	6 (10.7)
aortic root diameter, mm (range)	4.95 ± 0.55 (3.9–6.5)
relevant mitral regurgitation	14 (25)
bicuspid aortic valve	2 (3.6)
CAD	2 (3.6)
diabetes	0
hypertension	37 (66)
smoking	20 (36)
COPD	2 (3.6)
NYHA classification:	
I–II	43 (77)
III–IV	11 (20)
LV function:	
poor	0
moderate	4 (7.1)
good	52 (93)
Logistic EuroSCORE I	7.96 ± 5.2
creatinine, mg/dl	0.8 ± 0.6
bilirubin, mg/dl	0.74 ± 0.18
hyperlipidaemia	15 (27)
pulmonary hypertension	2 (3.6)
AAA	15 (27)
previous vascular intervention	4 (7.1)
renal impairment	0

Values are n (%) or mean ± SD. BMI: body mass index; CAD: coronary artery disease; COPD: chronic obstructive pulmonary disease; NYHA: New York Heart Association; LV: left ventricular; AAA: abdominal aortic aneurysm.

Intraoperative data are shown in Table 2. Nineteen patients (33.9%) underwent concomitant surgical procedures other than sole chest wall repair and direct patent foramen ovale closure. Except for pectus excavatum correction, all other procedures were performed while on CPB. Only two patients (3.6%) had a bicuspid aortic valve, of whom one patient needed triangular resection and cleft closure for optimal aortic valve function.

**Table 2:** Intraoperative data.

operation time, min	327 ± 71
CPB time, min	206 ± 54
cross-clamp time, min	148 ± 33
reperfusion time, min	45 ± 19
cardioplegia HTK/Bretschneider, ml	2259 ± 595
graft diameter, mm (median, range)	28 (18–38)
graft diameter/BSA, mm/m2	1.06
cusp intervention	2 (3.6)
concomitant procedures	26 (46.4%)
MV repair/replacement	13 (23.2)/1 (1.8)
CABG	0
TVR	0
aortic arch surgery	5 (8.9)
FET	1 (1.8)
cardiocirculatory arrest	5 (8.9)
cardiocirculatory arrest time, min (range)	39 (22–54)
chest wall procedure	2 (3.6)
patent foramen ovale closure	5 (8.9)
blood transfusion, U	1.23 ± 2.1
FFP, U	0.45 ± 1.1
PC, U	0.71 ± 0.96
CS blood, U	0.47 ± 0.97
IABP	0
catecholamines:	
norepinephrine	5 (8.9)
dobutamine	24 (43)
epinephrine	2 (3.6)
min. temperature, °C	33.75 ± 2.8

Values are n (%) or mean ± SD. CPB: cardiopulmonary bypass; BSA: body surface area; HTK: Histidine-tryptophan-ketoglutarate; MV: mitral valve; CABG: coronary artery bypass grafting; TVR: tricuspid valve repair; FET: frozen elephant trunk; FFP: fresh frozen plasma; PC: platelet concentrate; IABP: intra-aortic balloon pump.

Postoperative results are presented in Table 3. One patient (1.8%) needed re-exploration for bleeding on the day of surgery. One patient developed acute kidney injury requiring dialysis. We observed 2 AV blocks, with need of pacemaker implantation for complete AV block in 1 patient. Mean ICU stay was 1.38 ± 0.98 days followed by a mean of 3.7 ± 4.3 days on our intermediate care ward.

**Table 3:** Postoperative and follow-up data.

30-day/in-Hospital mortality	0
ICU stay, d (range)	1 (0–6)
respiratory insufficiency	0
ventilation time, h	14 ± 8.6
ARF	4 (7.1)
dialysis	1 (1.8)
dialysis at discharge	0
re-thoracotomy	1 (1.8)
blood transfusion, U	0.75 ± 1.38
FFP, U	0.23 ± 0.7
PC, U	0.39 ± 1.48
pericardial effusion	5 (8.9)
complete AV-block requiring pacemaker	1 (1.8)
hospital stay, d (range)	11 (6–47)
AR at discharge	trace to I°: 12; II°: 1
max. creatinine, mg/dl	0.76 ± 0.22
max. bilirubin, mg/dl	1.5 ± 1.2
phrenic injury	0
post MI	0
delirium	0
CV accident	0
follow-up, d	1858 ± 1161
AR II-III°	2 (3.6)
aortic valve reintervention	1 (1.8)
aortic dissection type B	1 (1.8)
aortic arch	1 (1.8)
descending/thoracoabdominal aortic replacement	2 (3.6)
infrarenal aortic replacement	2 (3.6)
FET	1 (1.8)
death during follow-up	0
pacemaker implantation during follow-up	0

Values are n (%) or mean ± SD. ICU: intensive care unit; ARF: acute renal failure; FFP: fresh frozen plasma; PC: platelet concentrate; AR: aortic regurgitation; MI: myocardial infarction; CV: cerebrovascular; FET: frozen elephant trunk.

As expected, patients undergoing concomitant procedures while being on-pump had significantly longer cross-clamp and bypass time (p < 0.001) and an increased need for intraoperative blood (p = 0.013) as well as platelet (p = 0.018) transfusion (Table 4). Furthermore, patients undergoing concomitant procedures needed significantly more postoperative blood transfusions (p = 0.011) (Table 4). Moreover, duration on ICU (p < 0.001) was significantly longer. However, additional surgical interventions had no influence on intubation time (p = 0.99) nor hospital stay (p = 0.203). Furthermore, no significant difference between postoperative maximum bilirubin (p = 0.456) or creatinine (p = 0.343) levels nor other postoperative complications were found. Regression analysis detected no influence of concomitant surgery on secondary aortic interventions during follow-up (p = 0.255).

**Table 4:** Influence of concomitant procedures.

David procedure (n = 56)	with concomitant procedure (19 patients)	with no concomitant procedure (37 patients)	*P* value
cross-clamp time, min	180 ± 28	133 ± 22	<0.001
bypass time, min	251 ± 55	182 ± 37	<0.001
intraoperative blood transfusion, U	2.25 ± 2.5	0.71 ± 1.61	0.013
postoperative blood transfusion, U	1.42 ± 1.87	0.41 ± 1.61	0.011
intubation time, h	17.8 ± 11.7	12.5 ± 5.7	0.99
ICU stay, d	2.0 ± 1.4	1.0 ± 0.37	<0.001
hospital stay, d	14 ± 9.2	11 ± 4.1	0.203
log. EuroSCORE I	9.2 ± 7.1	7.3 ± 3.8	0.111
postoperative max. bilirubin, mg/dl	1.17 ± 0.62	1.68 ± 1.44	0.456
postoperative max. creatinine, mg/dl	0.71 ± 0.14	0.79 ± 0.25	0.343
dialysis	1	0	0.159
reintervention of the aortic root during follow-up	1	0	0.159
reintervention of the aorta during follow-up	3	3	0.379

Values are n (%) or mean ± SD. ICU: intensive care unit.

During follow-up echocardiography, 24 patients (43%) showed signs of trivial to mild AR, one patient (1.8%) had moderate AR and another one (1.8%) presented with severe AR. That patient underwent re-do aortic valve replacement almost 3 years (1087 days) after initial surgery.

**Figure 1:**
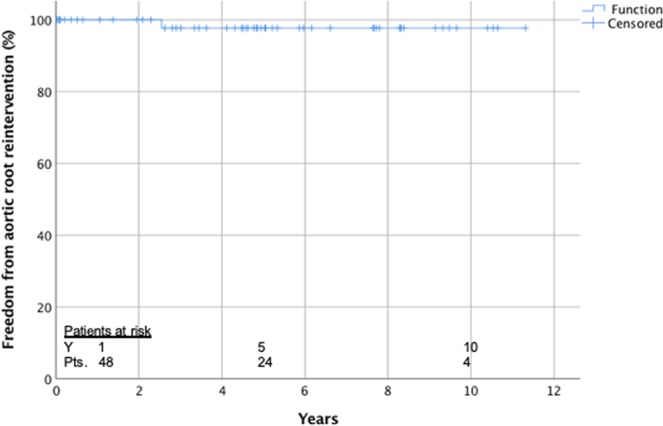
Actuarial freedom from reintervention of the aortic root.

**Figure 2:**
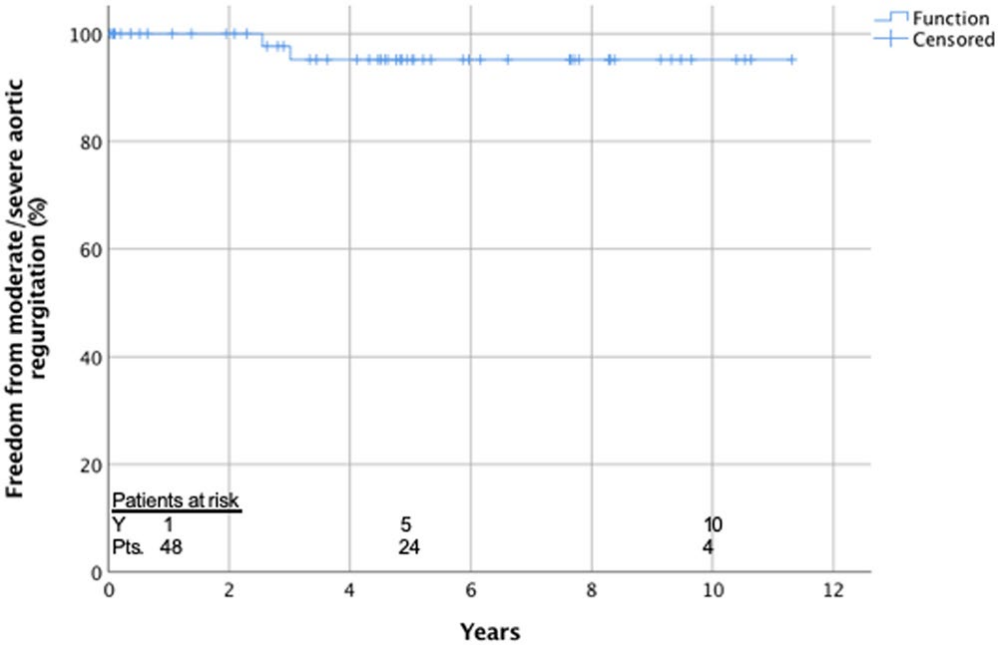
Actuarial freedom from moderate/severe aortic regurgitation.

Actuarial freedom of reintervention of the aortic root was 100% after 1 year and 97.7 ± 2.3 after 8 years (Fig. 1) and freedom of moderate and severe AR was 97.7 ± 2.3% after 1 and 95.2 ± 3.3% after 8 years (Fig. 2).

Reinterventions of the downstream aorta were necessary in 6 patients (10.7%). One open surgical repair for abdominal aortic aneurysm was performed 8 years after our initial surgery in another hospital, 2 patients underwent open thoraco-abdominal aortic surgery after 3 and 3.5 years respectively in our center. One patient first underwent aortic arch replacement for suture aneurysm with the elephant trunk technique one year after VSARR, then 3 weeks later received an open thoraco-abdominal vascular repair at our department. Due to Stanford type B dissection 4 years after VSARR, another patient was treated with the frozen elephant trunk technique in another hospital and one patient received endovascular aneurysm repair stent graft via bilateral transfemoral cut-down ipsilateral and iliac extension and contralateral extension from our vascular surgery department.

Regression analysis did not detect any significant predictive factors influencing early and long-term outcomes except for aortic dissection at the time of VSARR. Since 4 of the 6 patients who presented with aortic dissection at the time of operation were in need of reintervention of the downstream aorta during follow-up, a significant correlation was detected with a HR of 32.7 (CI 95% 3.6–294; p = 0.002). The entire list of analyzed parameters can be found as Supplementary Table S1.

## Discussion

Here, we present early and long-term results of VSARR using the reimplantation technique in patients with Marfan syndrome, over an 11-year-observation period at a single institution. Since it was first described by David *et al*.^3^., this technique has emerged as an invaluable treatment option for aortic root aneurysm repair^9^. Compared to the modified Bentall operation as the standard procedure for aortic root replacement, it offers various advantages such as evasion of life-long anticoagulation and reduced respective thromboembolic and endocarditis risks^10–13^. Hence, the therapeutic approach shifted more towards this valve-sparing technique, especially in this comparably young patient population, who in turn will benefit most from the aforementioned advantages^7,14^.

The valve-sparing aortic root replacement technique by David and associates has been performed as the standard root replacement procedure in patients with MFS at our center since 2007, by surgeons with a pre-existing experience in performing VSARR^4,7^. The benefit of this procedure over Yacoub’s remodeling technique is still a matter of ongoing debate. However, systematic reviews favor David’s method due to reduced annular dilation during follow-up^15^. Furthermore, this approach was reported to show superior outcome results in children^16^. This may be accounted for by the fixation of the Dacron tube to the aortic basis in the David technique, compared to an incomplete stabilization within the aortic annulus using Yacoub’s method. Similarly, the valve sparing technique described by Schäfers *et al*. using an additional annular stabilization suture has also been successfully applied in Marfan’s patients^17^. Hence, outcome analysis regarding long-term durability of this surgical approach is mandatory to further underline its value in this key demographic.

With no in-hospital mortality and only one patient undergoing re-operation of the aortic root during follow-up, our excellent results are comparable to recent reports^5,8,11,14^. In one of the longest published cohort studies, Martens *et al*. reported freedom of re-operation rates of 86% at 10 years and 80% at 20 years, in a single-center series of 104 MFS patients^8^. Kari *et al*. found that preoperative NYHA status and aortic size, were related to adverse outcome after VSARR concerning reintervention frequency^17^. In their study, the authors hypothesized that extensive aortic pathologies might lead to an excessive aortic downsizing during valve-sparing surgery. Thereby the risk of cusp billowing and prolapse later on, is enhanced consecutively. We did not observe the same effect, since only one patient developed AR requiring aortic valve replacement 51 month after initial surgery, despite the number of small grafts being used, especially in patients under 18 years of age. While Valsalva grafts are being utilized more frequently, almost all our patients received a classic straight tube graft, since the DAVID I procedure is the preferred technique at our center. The homogeneity of the procedure may further contribute to our excellent results.

Only 2 patients (3.6%) presented with acute aortic type A dissection and underwent emergency surgery. During follow-up, transthoracic echocardiography showed only trivial aortic valve regurgitations in both of these patients, 59 and 125 months after initial surgery, respectively. The other remaining 54 VSARR (96.4%) were elective aneurysmal aortic root replacements, which could have also contributed favorably to our outcomes. Furthermore, aortic dissection is a known risk factor with regard to reinterventions of the downstream aorta or the aortic root^18–20^. In our cohort operations of the aortic arch and descending aorta – including abdominal aorta - were performed in 6 patients, of whom 4 initially presented with aortic dissections (type A or B), which is also in line with previous findings^19–21^. This finding was further reflected in univariate Cox regression analysis, where the presence of aortic dissection showed an increased hazard for downstream aortic reinterventions.

During follow-up, no case of valvular endocarditis, or any adverse neurological events were recorded, which is in good standing with current literature^5,8^. Noteworthy is the observation that patients with MFS tend to develop at least one episode of postoperative blood-culture negative fever, without elevation of infectious parameters. This finding may be related to the enhanced inflammatory aspect of the aortic wall in MFS and is currently debated^22,23^. This phenomenon has been described after aortic surgery and may also be related to other factors such as endothelialization of the prosthesis^24^.

In general, oral anticoagulation regime with ASA 300 mg for 3 months was deemed sufficient to allow for endothelization of the Dacron graft and account for possible endovascular surface irregularities. Patients with concomitant MVR or otherwise existing indication for full anticoagulation, received oral Vitamin K Antagonist (Marcumar) to achieve target INR accordingly. Pericardial effusion was detected in 5 patients, which have been drained or conservatively treated by colchicine.

Due to the nature of the underlying disease, a substantial number of patients presented with the need for additional interventions at the time of surgery^8^. Despite the significantly longer cross-clamp time and increased amount of blood transfusion, concomitant on-pump procedures such as MVR and arch surgery did not negatively influence postoperative outcomes. Postoperative recovery was uneventful with no postoperative occurrences of cardiac low-output or need for mechanical circulatory support. Moreover, reintervention rates were similar when compared to isolated VSARR, a finding that has not yet been addressed in other reports. Adequate cardioprotective strategies, such as use of antegrade cardioplegic solution HTK/Bretschneider, local cooling and sufficient flushing of the coronary ostia, are key elements to achieve these results.

The overall low incidence of adverse clinical events in this patient’s cohort, is correlated to inherit patients’ demographic, such as young age and consecutively less encountered co-morbidities. As seen in our patients’ characteristics, the overall left ventricular function was preserved and most patients had a comparably high exercise capacity (NYHA I-II). Altogether determinants that have been previously identified as negative outcome predictors in other studies^14,17^.

Aortic root interventions in MFS patients are often necessary before the beginning of the 5^th^ decade, with the median age at the time of surgery becoming progressively younger due to the expanding indication for prophylactic operations. According to current guidelines, aortic surgery is recommended in all patients with MFS presenting at a maximal aortic diameter >50 mm^25^. With additional risk factors, such as family history of acute aortic dissections or an expected pregnancy, MFS patients should be considered for surgery even sooner (≥45 mm)^25^. Life expectancy of patients with MFS is now similar to that of the general population by reduction of lethal aortic complications, through the improved surgical results^26^.

Flynn *et al*. found in their systematic review of surgical outcomes in MFS patients undergoing aortic root surgery a reduced risk of thromboembolism, late hemorrhagic complications and endocarditis compared to composite-valve graft replacement^5^. More interestingly, they did not find a significant difference in reintervention rates between both surgical procedures, which was previously considered the limiting factor of the reimplantation technique, as reported by the meta-analysis of Benedetto *et al*.^19^. Our data further underline the role of VSARR procedure in this patient group, even if concomitant surgical procedures are necessary. However, it is elementary to know that not all patients are suitable for VSARR. Structural defects, such as leaflet fenestration or tissue redundancy, may prevent a satisfactory VSARR. The underlying connective tissue defect in MFS may also affect the aortic cusps and therefore compromise satisfactory long-term results. The expanding indication towards prophylactic surgery may further improve long-term results, since surgical intervention is performed at an earlier stage of the disease. Preserved aortic valve integrity and reduced co-morbidities are possible advantages, due to patient’s younger age.

Since only two patients presented with bicuspid aortic valves in our study cohort, a general recommendation for VSARR in these patients cannot be given. However, both patients showed no aortic regurgitation, nor structural valve disease. They are both in NYHA class I after a follow-up period of 118 and 129 months respectively. Thus, an individual case by case decision should be undertaken in these cases.

We emphasize the importance of dedicated MFS centers performing David’s procedure, where competence and experience are concentrated. Our patients are monitored in our MFS clinic with regular MRA imaging of the aorta to avoid radiation. Follow-up is complete and long-term results can be collected hereby. Being able to treat MFS patients in a highly experienced, high-volume center allows complete understanding of the individual aortic pathology. It is well known that phenotypical differences as well as varying vascular involvement of MFS are present in this heterogenic patient cohort.

This study is limited by its retrospective design and thus succumbs to inherit bias. Due to the cohort size and lack of adverse events, extensive regression analysis is not possible. Moreover, we did not compare outcomes to a reference group of modified Bentall procedures, since only few patients with definitive diagnosis of MFS underwent this procedure, making a statistical analysis unfeasible.

## Conclusion

In conclusion, we strongly recommend VSARR using David’s procedure for aortic root pathologies and prophylactic surgery in patients with MFS, even if complex concomitant procedures are needed, delivering excellent early and long-term results, especially in terms of freedom from significant aortic regurgitation, thromboembolic events, reintervention and endocarditis.

## Data Availability

All relevant data are included within the manuscript.

## Supplementary information

Supplementary Table S1

## References

[1] Robinson PN (2006). The molecular genetics of Marfan syndrome and related disorders. J Med Genet.

[2] Loeys BL (2010). The revised Ghent nosology for the Marfan syndrome. J Med Genet.

[3] David, T. E. & Feindel, C. M. An aortic valve-sparing operation for patients with aortic incompetence and aneurysm of the ascending aorta. *J Thorac Cardiovasc Surg* **103**, 617–621; discussion 622 (1992). 1532219

[4] Kallenbach Klaus, Baraki Hassina, Khaladj Nawid, Kamiya Hiroyuki, Hagl Christian, Haverich Axel, Karck Matthias (2007). Aortic Valve–Sparing Operation in Marfan Syndrome: What Do We Know After a Decade?. The Annals of Thoracic Surgery.

[5] Flynn CD (2017). Systematic review and meta-analysis of surgical outcomes in Marfan patients undergoing aortic root surgery by composite-valve graft or valve sparing root replacement. Ann Cardiothorac Surg.

[6] David TE (2015). Outcomes of Aortic Valve-Sparing Operations in Marfan Syndrome. J Am Coll Cardiol.

[7] Karck M (2004). Aortic root surgery in Marfan syndrome: Comparison of aortic valve-sparing reimplantation versus composite grafting. J Thorac Cardiovasc Surg.

[8] Martens A (2018). Valve-sparing aortic root replacement (David I procedure) in Marfan disease: single-centre 20-year experience in more than 100 patients. Eur J Cardiothorac Surg.

[9] Miller DC (2003). Valve-sparing aortic root replacement in patients with the Marfan syndrome. J Thorac Cardiovasc Surg.

[10] Baumgartner, W. A., Cameron, D. E., Redmond, J. M., Greene, P. S. & Gott, V. L. Operative management of Marfan syndrome: The Johns Hopkins experience. *Ann Thorac Surg* **67**, 1859–1860; discussion 1868–1870 (1999). 10.1016/s0003-4975(99)00412-9 10391326

[11] Coselli JS, Weldon SA, Preventza O, de la Cruz KI, LeMaire SA (2017). Valve-sparing versus composite root replacement procedures in patients with Marfan syndrome. Ann Cardiothorac Surg.

[12] Gott VL (1999). Replacement of the aortic root in patients with Marfan’s syndrome. N Engl J Med.

[13] Bernhardt AM (2011). Comparison of aortic root replacement in patients with Marfan syndrome. Eur J Cardiothorac Surg.

[14] Price J (2016). Long-term outcomes of aortic root operations for Marfan syndrome: A comparison of Bentall versus aortic valve-sparing procedures. J Thorac Cardiovasc Surg.

[15] Tian D, Rahnavardi M, Yan TD (2013). Aortic valve sparing operations in aortic root aneurysms: remodeling or reimplantation?. Ann Cardiothorac Surg.

[16] Patel Nishant D., Arnaoutakis George J., George Timothy J., Allen Jeremiah G., Alejo Diane E., Dietz Harry C., Cameron Duke E., Vricella Luca A. (2011). Valve-sparing aortic root replacement in children: intermediate-term results☆. Interactive CardioVascular and Thoracic Surgery.

[17] Kari FA (2014). David I reimplantation procedure for aortic root replacement in Marfan patients: medium-term outcome. Interact Cardiovasc Thorac Surg.

[18] Benedetto U (2011). Surgical management of aortic root disease in Marfan syndrome: a systematic review and meta-analysis. Heart.

[19] Puluca N, Burri M, Cleuziou J, Krane M, Lange R (2018). Consecutive operative procedures in patients with Marfan syndrome up to 28 years after initial aortic root surgery. Eur J Cardiothorac Surg.

[20] Rylski B (2014). Type A aortic dissection in Marfan syndrome: extent of initial surgery determines long-term outcome. Circulation.

[21] Schoenhoff FS, Carrel TP (2017). Re-interventions on the thoracic and thoracoabdominal aorta in patients with Marfan syndrome. Ann Cardiothorac Surg.

[22] Arif R (2017). AP-1 Oligodeoxynucleotides Reduce Aortic Elastolysis in a Murine Model of Marfan Syndrome. Mol Ther Nucleic Acids.

[23] Seppelt PC (2016). Loss of Endothelial Barrier in Marfan Mice (mgR/mgR) Results in Severe Inflammation after Adenoviral Gene Therapy. PLoS One.

[24] Yao YT (2009). Noninfectious fever following aortic surgery: incidence, risk factors, and outcomes. Chin Med Sci J.

[25] Baumgartner H (2017). ESC/EACTS Guidelines for the management of valvular heart disease. Eur Heart J.

[26] Silverman DI (1995). Life expectancy in the Marfan syndrome. Am J Cardiol.

