# Supplementary material for: The reimplantation valve-sparing aortic root replacement technique for patients with Marfan syndrome: A single-center experience
Source: Sci Rep. 2019 Aug 19;9:12021. doi: 10.1038/s41598-019-48572-9 (PMC6700152; doi:10.1038/s41598-019-48572-9)
Supplement: Supplementary file 1 — Supplementary Table S1 [file 41598_2019_48572_MOESM1_ESM.docx]

**The reimplantation valve-sparing aortic root replacement technique for patients with Marfan syndrome: A single-center experience.**

Jamila Kremer^1*^; Mina Farag^1*^; Marcin Zaradzki^1^; Gabor Szabó^1^; Arjang Ruhparwar^1^; Klaus Kallenbach^1, 2^; Matthias Karck^1^; Rawa Arif^1^

*both authors contributed equally

^1^Marfan Center University Hospital Heidelberg, Department of Cardiac Surgery, University Hospital Heidelberg, Germany

^2^INCCI HaerzZenter, Department of Cardiac Surgery, Luxembourg, Luxembourg

Running Title: David Procedure for Marfan Syndrome

**Corresponding Address:**

Dr. med. Rawa Arif

Department of Cardiac Surgery

University Hospital Heidelberg

Im Neuenheimer Feld 110

69120 Heidelberg, Germany

e-mail: [rawa.arif@med.uni-heidelberg.de](mailto:rawa.arif@med.uni-heidelberg.de)

phone: 0049 (0) 6221 56 6882

fax: 0049 (0) 6221 56 5585

**Supplementary Table S1**

Cox regression analysis for identification of risk factors for secondary aortic intervention.

| risk factors | univariate logistic regression | |
| --- | --- | --- |
|  | *P* value | HR (95% CI) |
| age | 0.240 |  |
| Logistic EuroSCORE I | 0.853 |  |
| gender | 0.996 |  |
| BMI | 0.225 |  |
| LV function | 0.664 |  |
| previous vascular surgery | 0.073 |  |
| aortic cross-clamp time | 0.134 |  |
| blood transfusion | 0.416 |  |
| concomitant procedures | 0.255 |  |
| aortic dissection at time of operation | 0.002 | 32.665 (3.631 – 294.848) |

BMI: body mass index; LV: left ventricular.
